# A Pair of Maternal Chromosomes Derived from Meiotic Nondisjunction in Trisomy 21 Affects Nuclear Architecture and Transcriptional Regulation

**DOI:** 10.1038/s41598-017-00714-7

**Published:** 2017-04-10

**Authors:** Sayaka Omori, Hideyuki Tanabe, Kimihiko Banno, Ayumi Tsuji, Nobutoshi Nawa, Katsuya Hirata, Keiji Kawatani, Chikara Kokubu, Junji Takeda, Hidetoshi Taniguchi, Hitomi Arahori, Kazuko Wada, Yasuji Kitabatake, Keiichi Ozono

**Affiliations:** 1 Department of Pediatrics, Graduate School of Medicine, Osaka University, Suita, Osaka 565-0871 Japan; 2 Japan Society for the Promotion of Science, Chiyoda-ku, Tokyo 102-0083 Japan; 3 Department of Evolutionary Studies of Biosystems, School of Advanced Sciences, SOKENDAI (The Graduate University for Advanced Studies), Shonan Village, Hayama, Kanagawa 240-0193 Japan; 4 Department of Genome Biology, Graduate School of Medicine, Osaka University, Suita, Osaka 565-0871 Japan

## Abstract

Eukaryotic genomes are organised into complex higher-order structures within the nucleus, and the three-dimensional arrangement of chromosomes is functionally important for global gene regulation. The existence of supernumerary chromosome 21 in Down syndrome may perturb the nuclear architecture at different levels, which is normally optimised to maintain the physiological balance of gene expression. However, it has not been clearly elucidated whether and how aberrant configuration of chromosomes affects gene activities. To investigate the effects of trisomy 21 on nuclear organisation and gene expression, we performed three-dimensional fluorescent imaging analysis of chromosome-edited human induced pluripotent stem cells (iPSCs), which enabled identification of the parental origin of the three copies of chromosome 21. We found that two copies of maternal chromosomes resulting from meiotic nondisjunction had a higher tendency to form an adjacent pair and were located relatively distant from the nuclear membrane, suggesting the conserved interaction between these homologous chromosomes. Transcriptional profiling of parental-origin-specific corrected disomy 21 iPSC lines indicated upregulated expression of the maternal alleles for a group of genes, which was accompanied by a fluctuating expression pattern. These results suggest the unique effects of a pair of maternal chromosomes in trisomy 21, which may contribute to the pathological phenotype.

## Introduction

Gene activities are not only determined by *cis-* and *trans-*acting regulatory DNA sequences, but are also dynamically regulated by epigenetic events including DNA methylation, histone modifications and chromatin remodelling^1–3^. In addition to these direct alterations of chromatin configuration, accumulating evidence suggests that the higher-order chromosome organisation in the nucleus participates in the epigenetic control of gene expression^4^. During interphase, chromosomes occupy discrete and mutually exclusive fraction of the nuclear volume and represent structural units called chromosome territories (CTs)^5, 6^. These CTs are nonrandomly arranged within the nuclear space in a tissue-specific manner^7, 8^ and, as reported previously, the radial arrangement of CTs is evolutionarily conserved in the genomes of higher primates^9^. Although CTs are discrete structures that seem to remain stable during interphase, some loci are dynamically relocalised depending on the transcriptional status. Transcriptionally active genes tend to be repositioned at the periphery of their CT, which may allow the genes to access the transcriptional machinery termed transcription factories^10^. This nuclear architecture is closely related to global gene expression, and some studies have suggested that structural defects in higher-order chromatin and chromosome organisation underlie certain disease mechanisms^11, 12^.

Down syndrome (DS, trisomy 21) is the most common form of chromosomal aneuploidy, which results from an extra copy of human chromosome 21 (HSA21)^13, 14^. Individuals with DS have a wide range of medical conditions including intellectual disability, congenital heart defects and early onset of Alzheimer’s disease^15^. Each disease phenotype is generally thought to be derived from the increased expression of a specific subset of dosage-sensitive genes, and several analyses of gene expression profiling have revealed that most of the triplicated genes showed upregulated expression consistent with gene dosage (i.e., a 1.5-fold increase)^16–19^. However, the transcriptional levels of some genes were found not to be directly proportional to the number of copies, and showed upregulation, compensation or downregulation in a tissue-specific manner. Although such inconsistency in gene expression patterns could contribute to the complex and highly variable pathological phenotypes observed in DS patients, the details of the regulatory mechanisms of gene expression in trisomy cells have not been clearly demonstrated.

Here, we investigated the parental-origin-specific effects on chromosome organisation and transcriptional regulation in trisomy 21. We successfully distinguished the one paternal chromosome from the two copies of the maternal chromosome and identified their localisation patterns in the nucleus, using an artificially generated partial trisomy induced pluripotent stem cell (iPSC) line. We further introduced targeted chromosome elimination using chromosome-editing technologies and established three corrected disomy 21 iPSC lines in which each one of the three copies of chromosome 21 was selectively eliminated from the trisomy 21 iPSCs. Fluorescent imaging analysis of these iPSCs demonstrated distinctive positional patterns of the three copies of chromosome 21 in the nucleus, which suggested the sustained association of the two maternal chromosomes derived from meiotic nondisjunction. Intriguingly, gene expression profiling of these iPSC lines demonstrated that there were several genes whose expression levels were significantly upregulated only in the maternal alleles, despite the almost equal amounts of transcripts among each allele for other genes. Our study provides a unique insight into the chromosome organisation and gene regulation in trisomy 21.

## Results

### Two Copies of Maternal Chromosome 21 Show a Characteristic Positional Pattern in the Nucleus of Trisomy iPSCs

**Figure 1:**
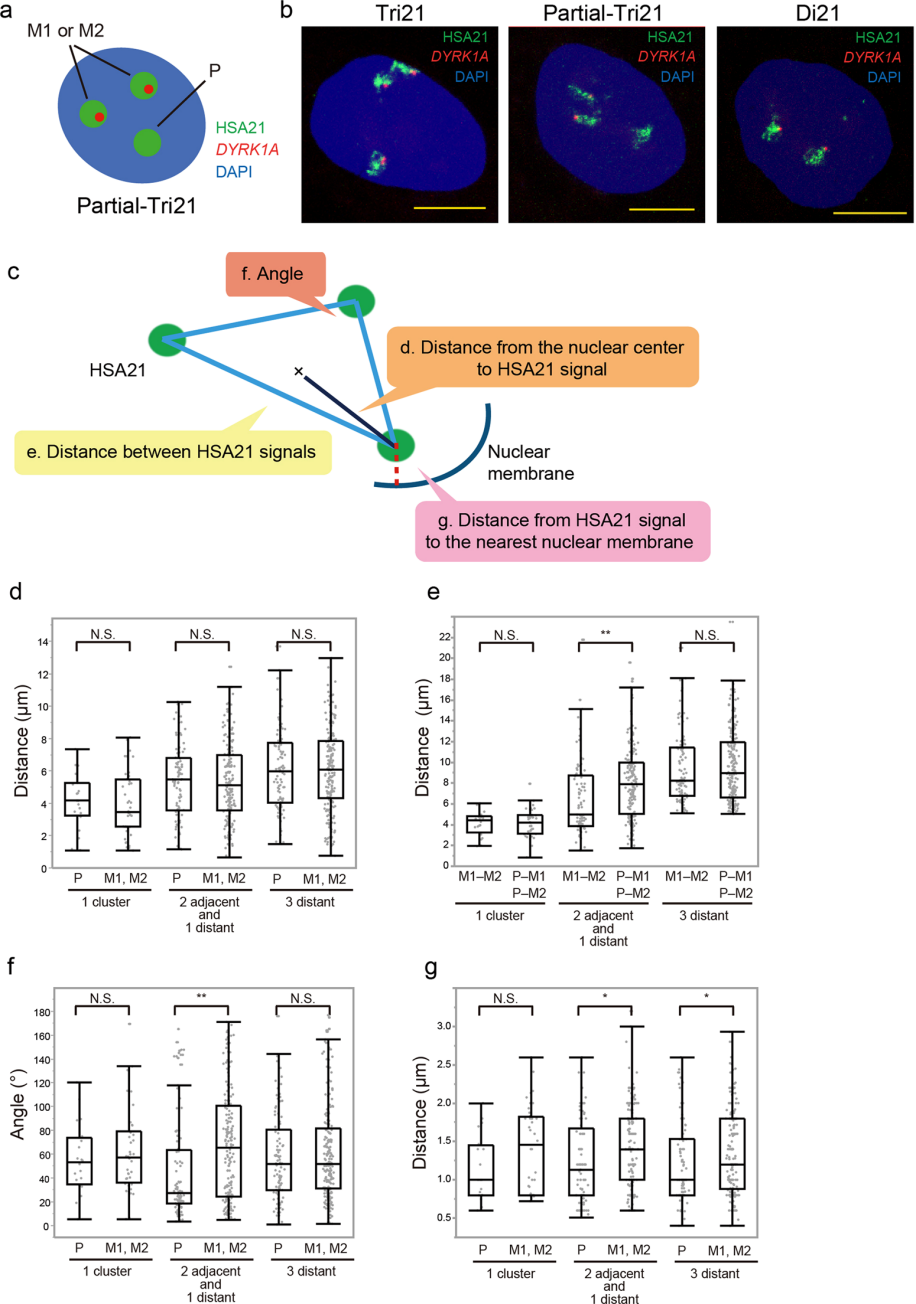
Chromosome positioning of paternal and maternal chromosome 21 in Partial-Tri21 iPSCs. (**a**) A schematic of the 3D-FISH analysis in Partial-Tri21 iPSCs. Signals of chromosome 21 (green) with or without a *DYRK1A* signal (red) represent chromosomes of maternal (M1/M2) or paternal origin, respectively. (**b**) Representative 3D-FISH images of Tri21, Partial-Tri21 and Di21 iPSCs. Chromosome 21 was labelled with Alexa488 (green), while the *DYRK1A* gene was labelled with Cy3 (red). Nuclei were stained with 4′,6-diamidino-2-phenylindole (DAPI). Scale bars represent 10 μm. (**c**) A schematic representation of the distance measurements in 3D-FISH analysis. (**d–g**) Localisations of paternal and maternal chromosome 21 in Partial-Tri21 iPSCs. Localisation patterns of chromosome 21 categorised into three groups: 1 cluster (n = 20), 2 adjacent and 1 distant (n = 80), and all distant (n = 99). Measurements included (**d**) the distance from the nuclear centre to each copy of chromosome 21, (**e**) the distance between two copies of chromosome 21, (**f**) interior angles at the vertex and (**g**) the distance from chromosome 21 to the nearest nuclear membrane. The term “adjacent” here refers to the distance between copies of chromosome 21 being under 5 μm. Box plot represents 25th–75th percentile range ± min–max. p values were determined by the Mann–Whitney U test. *p < 0.05, **p < 0.01.

In nuclei of the lymphocyte cell lines derived from individuals with DS, two copies of chromosome 21 were reported to localise proximal to one another but far from the third chromosome^20^. To study whether the parental origin of the particular copies of chromosome 21 is relevant to CTs in the trisomy cells, we performed three-dimensional fluorescence *in situ* hybridisation (3D-FISH) analysis of human iPSCs in combination with genome-editing technology to identify the parental origin of the three chromosomes. We previously generated a patient-derived trisomy 21 iPSC (‘Tri21-GATA1wt’ iPSC) line that contains one paternal and two maternal copies of chromosome 21 (Supplementary Fig. S1), and a partial trisomy 21 iPSC (‘Partial-Tri21-GATA1wt’ iPSCs) line (hereafter referred to as ‘Tri21’ iPSCs and ‘Partial-Tri21’ iPSCs, respectively)^17^. In this Partial-Tri21 iPSC line, a 4-Mb region on HSA21 was selectively deleted only from the paternal chromosome 21 in Tri21 iPSCs, so these genetically modified cells can be used to clarify the parental origin of chromosome 21. 3D-FISH analysis using a probe specific to chromosome 21 showed that the positional patterns of three signals of chromosome 21 were distinct in human iPSCs and similar in the two iPSC lines mentioned above (Supplementary Fig. S2a). Measurements of the nuclear volume, the distance from the nuclear centre to the chromosome signals, the distance between each copy of chromosome 21, the interior angles in the triangle formed by the three signals and the distance from the signal to the nearest nuclear membrane revealed that the values in Partial-Tri21 iPSCs were nearly identical to those in original Tri21 iPSCs (Supplementary Fig. S2b–f), suggesting that the deletion of the 4-Mb region caused no distinct change in the CTs of chromosome 21 in trisomic iPSCs. Double labelling with a probe for the *DYRK1A* gene, which is located inside the 4-Mb region and was deleted only from the paternal chromosome in the Partial-Tri21 iPSC line, enabled identification of the parental origin of chromosome 21 (Fig. 1a,b). Regarding the distance from the nuclear centre to each signal, there were no differences between paternal and maternal chromosomes (Fig. 1c,d). Interestingly, among the cell group with the pattern of two adjacent and one isolated signals, approximately 48% of cells showed the combination of an adjacent pair of chromosome 21 copies of maternal origin and an isolated single chromosome of paternal origin. This proportion is higher than would be expected if the positioning were random (i.e., 33.3%), suggesting that the retained maternal chromosomes resulting from meiotic nondisjunction may conserve their interaction and affect the CTs in the nucleus. Reflecting this positional pattern, in the triangle formed by the three signals, the side length between the two maternal chromosomes was significantly shorter than the other two sides that included the paternal chromosome (Fig. 1e). Similarly, the interior angles at the vertex with the paternal signal were smaller than those at the other vertices with the maternal signals (Fig. 1f). There were no significant differences in the nuclear volumes between the cell group that showed the pattern of two adjacent and one distant signals of chromosome 21 and the cell group with all distant signals (1195 ± 429 μm^3^ and 1214 ± 314 μm^3^, respectively). In addition, in the cells with the pattern of two adjacent and one isolated signals, the nuclear sizes were consistent between the cell group with an isolated single chromosome of paternal origin and the other group containing an isolated signal of maternal origin (1192 ± 321 μm^3^ and 1106 ± 365 μm^3^, respectively). This rules out the possibility that the alteration of nuclear size affects the chromosome positioning pattern. Notably, the distance from each maternal signal to the nearest nuclear membrane was significantly greater than that from the paternal signal (Fig. 1g). These results suggest that the nuclear localisation of a paternal chromosome and that of a pair of maternal chromosomes derived from nondisjunction are differentially regulated in trisomy 21.

### Targeted Correction of Trisomy 21 in Human iPSCs Rescues Aberrant Cellular Phenotypes

**Figure 2:**
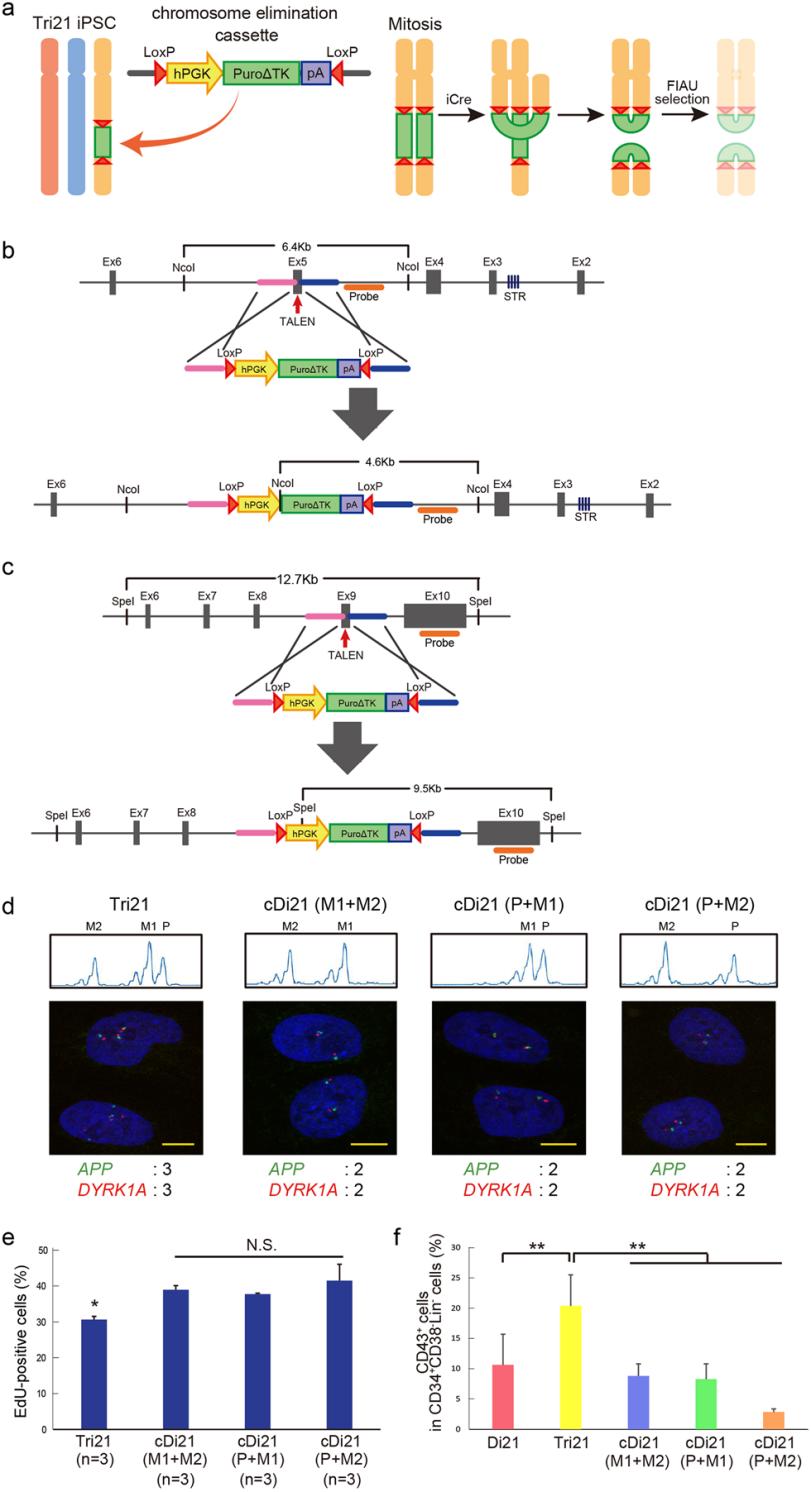
Establishment of corrected Di21 iPSCs. (**a**) Method for establishing corrected Di21 (cDi21) iPSCs using chromosome elimination technology. Chromosome elimination cassette was introduced into one of three copies of chromosome 21 in Tri21 iPSCs by TALENs. Cre–loxP-mediated recombination between sister chromatids in mitosis caused dicentric chromosomes and those without centromeres. Daughter cells without these aberrant chromosomes were obtained using FIAU selection. (**b,c**) Targeting strategy for introducing chromosome elimination cassette into (**b**) *RUNX1* and (**c**) *ETS2* loci on chromosome 21. Enlarged illustrations indicate each gene locus, including exons (grey boxes), TALEN target sites (red arrows), microsatellite sequences (purple stripe), homology arms (pink and blue bars) and probes for Southern blot analysis (orange bars). Below are illustrations of each donor plasmid including homology arms, human PGK promoters, drug-resistance genes (PuroΔTK), poly(A) sequences and loxP sequences (red triangles). PuroΔTK, fusion protein of puromycin and a truncated version of herpes simplex virus type 1 thymidine kinase. In targeting *RUNX1* and *ETS2* genes, STR and SNP analyses were used, respectively, to identify the parental origin of chromosome 21. (**d**) Top: Short tandem repeat (STR) analysis in each human iPSC (hiPSC) line. STR analysis of the patient with DS and his parents was performed to identify each allele of the *RUNX1* gene in Tri21 iPSCs. STR sequences of *RUNX1* were compared between Tri21- and each cDi21-iPSCs. Bottom: DNA FISH of chromosome 21-specific probes. *APP* gene was labelled with DyLight488 (green) and *DYRK1A* gene was labelled with DyLight594 (red) probe. Nuclei were stained with DAPI. Expected numbers of *APP* and *DYRK1A* foci in each iPSC line are shown below. cDi21(M1 + M2) and cDi21(P + M1) were established through *RUNX1* targeting, while cDi21(P + M2) was established through *ETS2* targeting. Scale bars represent 10 μm. (**e**) EdU cell proliferation assay in each hiPSC line. Graph shows the proportion of EdU-positive cells. (**f**) Haematopoietic differentiation of each cell line. Graph shows the proportion of CD43-positive cells in CD34^+^CD38^−^Lin^−^ cells derived from day 8 embryoid bodies (n = 3). Error bars represent SEM. p values were determined by Student’s t-test. *p < 0.05, **p < 0.01.

To study the different cellular function of three homologous chromosomes separately, we generated three types of corrected disomy 21 (cDi21) iPSC in which each copy of chromosome 21 was selectively eliminated from the original trisomic cells. Based on the chromosome elimination technique in mouse embryonic stem cells^21^, we constructed a modified chromosome elimination cassette containing a puromycin-delta thymidine kinase (puroΔTK) flanked by inverted loxP sites (Fig. 2a). This cassette was integrated into the *RUNX1* or *ETS2* locus in Tri21 iPSCs using transcription activator-like effector nucleases (TALENs) (Fig. 2b,c). Puromycin-resistant iPSC clones were isolated and analysed by short tandem repeat (STR) and single-nucleotide polymorphism (SNP) genotyping to identify the parental origin of the targeted allele. Homologous recombination was verified by Southern blot analysis (Supplementary Fig. S3a,b). Three types of clone, in which the puroΔTK cassette had been inserted into only a single allele of *RUNX1* or *ETS2*, were subjected to Cre recombinase-mediated excision followed by 1-[2-deoxy-2-fluoro-8-D-arabinofuranosyl]-5-iodouracil (FIAU) negative selection (Fig. 2a). Elimination of one copy of chromosome 21 and its origin were confirmed by STR analysis, DNA FISH and karyotype analysis (Fig. 2d and Supplementary Fig. S3c). Hereafter, these cell lines are referred to as ‘cDi21(M1 + M2)’, ‘cDi21(P + M1)’ and ‘cDi21(P + M2)’ iPSCs.

To assess whether targeted correction of trisomy 21 can rescue the cellular phenotype, these corrected disomy 21 iPSC lines were subjected to a phenotype assay. Several studies have reported that cell proliferation was impaired in trisomy cells^22^. Cell proliferation assay using trisomy and corrected disomy cells revealed that proliferation impairments were significantly ameliorated in all of the corrected disomy 21 iPSC lines (Fig. 2e). Next, we differentiated the healthy control disomy 21 (Di21), trisomy 21 (Tri21) and corrected disomy 21 iPSC lines into haematopoietic lineages to evaluate their haematopoietic differentiation potential. Recently, we have reported that trisomy 21 augments foetal haematopoiesis through the acceleration of early haematopoietic commitment^17^. The proportion of CD43^+^ cells in the CD34^+^CD38^−^Lin^−^ fraction, which represents committed haematopoietic progenitors, was significantly increased in the Tri21 iPSC line compared with that in the Di21 iPSC lines, as reported previously (Fig. 2f). Notably, the proportion of CD43^+^ cells in the corrected disomy 21 iPSC lines was significantly lower than that in the Tri21 iPSC line and returned to the normal level observed in the Di21 iPSC line. These results suggest that correction of trisomy 21 using targeted chromosomal elimination technology can rescue cellular phenotypes caused by chromosomal aneuploidy.

### Positional Pattern of Maternal Chromosome 21 is Preserved in Corrected Disomy 21 Cells

**Figure 3:**
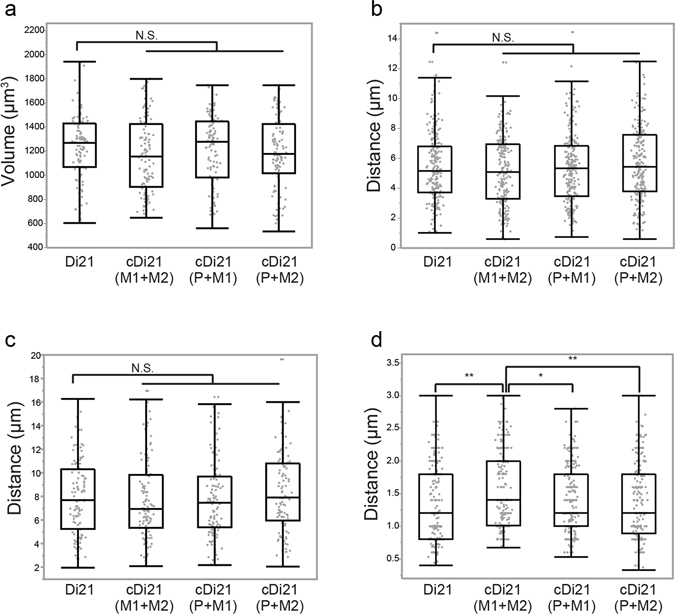
Positioning of chromosome 21 in Di21 and cDi21 iPSCs. (**a–d**) Chromosomal localisation in each hiPSC line [Di21, n = 110; cDi21(M1 + M2), n = 110; cDi21(P + M1), n = 116; cDi21(P + M2), n = 108]. Measurements included (**a**) the nuclear volume, (**b**) the distance from the nuclear centre to each copy of chromosome 21, (**c**) the distance between two copies of chromosome 21 and (**d**) the distance from chromosome 21 to the nearest nuclear membrane. Box plot represents 25th–75th percentile range ± min–max. p values were determined by the Mann–Whitney U test. *p < 0.05, **p < 0.01.

To study whether the distinct localisation pattern of trisomic chromosomes in the nucleus was preserved in the corrected disomy 21 cells, these iPSCs were subjected to 3D-FISH analysis. Detection with a probe specific to chromosome 21 demonstrated that the proportion of the cells in which two signals of chromosome 21 form one cluster was slightly higher in cDi21(M1 + M2) iPSCs than that in cDi21(P + M1) and cDi21(P + M2) iPSCs, although this was not statistically significant (22.7%, 19.0% and 18.5%, respectively). There were no differences in the nuclear volume, the distance from the nuclear centre to the chromosome signals and the chromosomal distances between the fluorescence centres among the iPSC control and corrected disomy 21 lines (Fig. 3a–c). On the other hand, although there were no significant differences in the distance from the signal to the nearest nuclear membrane among Di21, cDi21(P + M1) and cDi21(P + M2) iPSCs, the distance for cDi21(M1 + M2) iPSCs was significantly greater than that for the other iPSC lines (Fig. 3d). These results, together with the data for Partial-Tri21 iPSCs, indicate that a pair of maternal chromosomes caused by meiotic nondisjunction have a unique positional pattern in the nucleus.

### Parental Origin of Chromosome 21 Does Not Affect Nuclear Positioning in Partial Monosomy 21 Cells

**Figure 4:**
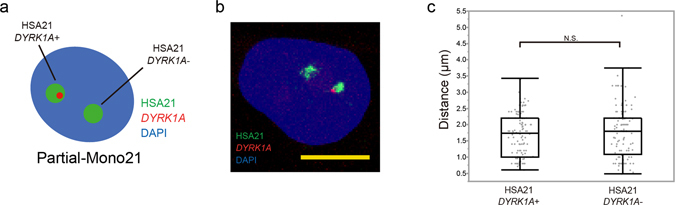
Chromosome positioning of chromosome 21 in Partial-Mono21 iPSCs. (**a**) A schematic of 3D-FISH analysis in Partial-Mono21 iPSCs. Two copies of chromosome 21 (green) can be distinguished each other by co-labelling with a *DYRK1A* probe (red). (**b**) Representative 3D-FISH images of Partial-Mono21 iPSCs. Chromosome 21 was labelled with Alexa488 (green), while the *DYRK1A* gene was labelled with Cy3 (red). Nuclei were stained with DAPI. Scale bars represent 10 μm. (**c**) The distance from chromosome 21 to the nearest nuclear membrane in Partial-Mono21 iPSCs (n = 120). Box plot represents 25th–75th percentile range ± min–max. p values were determined by the Mann–Whitney U test. *p < 0.05, **p < 0.01.

To investigate whether the parental origin of chromosome 21 affects nuclear localisation and causes differences in positioning pattern between a paternal copy and a pair of maternal copies of chromosome 21, another iPSC line was generated from dermal fibroblasts derived from a 13-year-old patient with partial monosomy of chromosome 21^23^. Although the total number of chromosomes was normal in this patient, a small region of 480 kb in size including *DYRK1A* was deleted from only a single copy of chromosome 21 (hereafter referred to as ‘Partial-Mono21’ iPSCs). Double labelling in 3D-FISH analysis with the probes for *DYRK1A* and chromosome 21 in this cell line enabled us to discriminate one copy of chromosome 21 from another (Fig. 4a,b). Probably because of the differences of original cell characteristics, the nuclear volume of Partial-Mono21 iPSCs, which were derived from the fibroblasts of the adolescent patient, was smaller than that of other cord blood cell-derived iPSCs (859.1 ± 242 μm^3^ in Partial-Mono21 iPSCs, see also Figs 3a and S2b). Because of this difference, a simple comparison of the signal distances between those different iPSC lines was inappropriate. However, in the same nucleus of Partial-Mono21 iPSCs, there were no significant differences in the distance from the signal to the nearest nuclear membrane between chromosome 21 with and without the *DYRK1A* signal, suggesting that the parental origin of chromosome 21 does not markedly affect its localisation pattern, at least in disomy cells (Fig. 4c).

### A Group of Genes on Chromosome 21 is Transcriptionally Upregulated in Maternal Alleles

**Figure 5:**
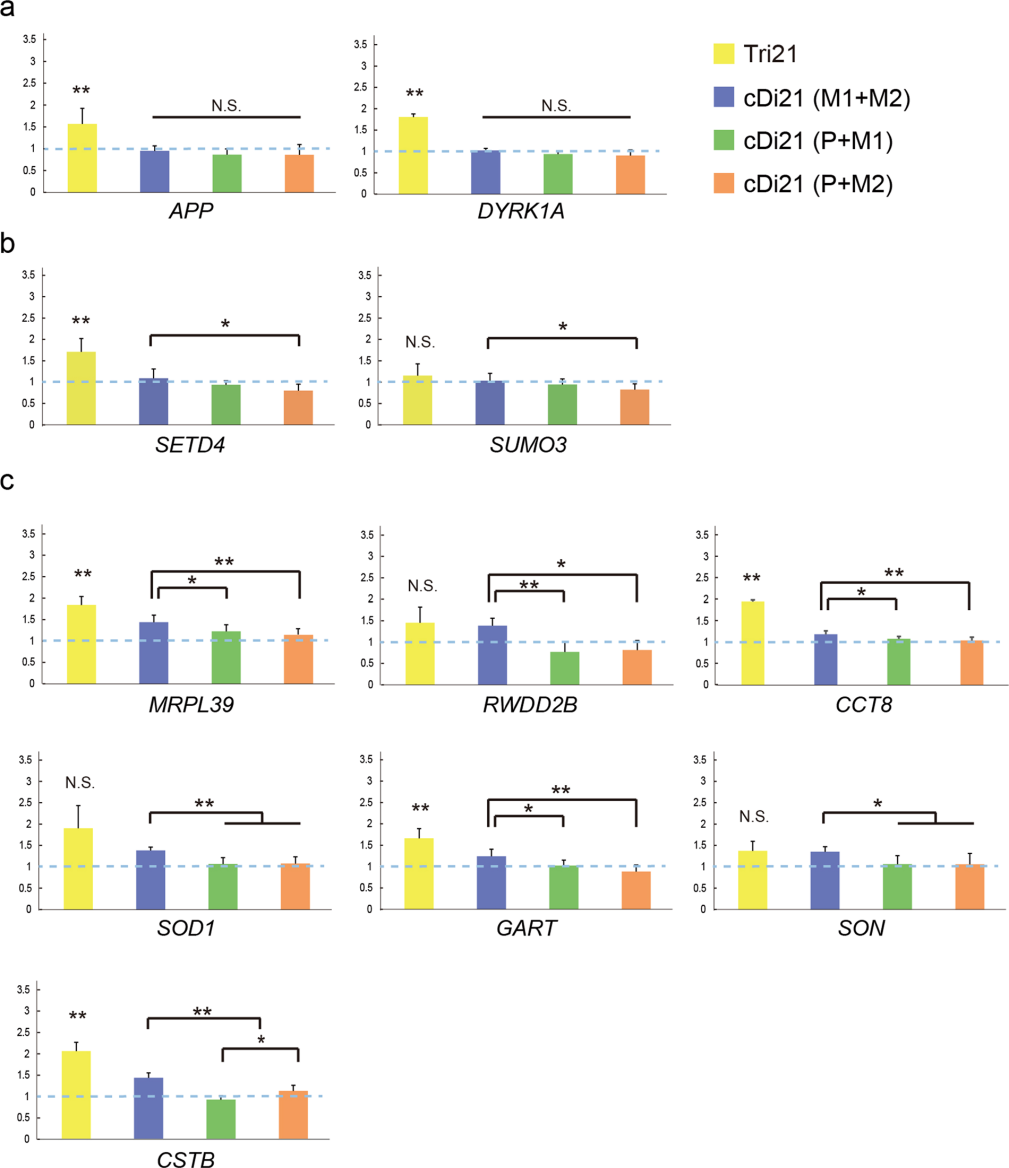
Gene expression analysis in Tri21 and cDi21 iPSCs. Relative expression levels of genes on chromosome 21 in Tri21 (yellow, n = 3), cDi21(M1 + M2) (blue, n = 9), cDi21(P + M1) (green, n = 9) and cDi21(P + M2) (orange, n = 9) iPSCs. Gene expression was normalised to that of Di21. (**a**) Genes whose expression levels did not differ markedly between cDi21 iPSCs (see also Supplementary Fig. S5). (**b**) Genes whose expression levels were higher in cDi21(M1 + M2) than in cDi21(P + M2) (see also Supplementary Fig. S6). (**c**) Genes whose expression levels were higher in cDi21(M1 + M2) than in cDi21(P + M1) and cDi21(P + M2). Error bars represent SEM. p values were determined by Student’s t-test or the Mann–Whitney U test. *p < 0.05, **p < 0.01.

To investigate whether maternal and paternal alleles of trisomic chromosomes have differences in their function, we analysed the gene expression profile in the chromosome-edited iPSCs lines. Among approximately 330 genes coded on chromosome 21, we focused on the genes whose absolute expression levels in disomy 21 iPSCs were moderate to high, and for which the expression levels in trisomy cells were upregulated above those in disomy cells. Based on the microarray data of our own experiment and a previous report^22^, 49 genes were selected (Supplementary Fig. S4) and their relative expression in Tri21, cDi21(M1 + M2), -(P + M1), and -(P + M2) iPSC lines compared with that in Di21 iPSCs was quantified by qRT-PCR. The expression levels in Tri21 iPSCs were upregulated for all of the genes and significant differences were observed for 33 genes. There were no differences among the corrected disomy 21 iPSC lines in 35 genes, including *APP* and *DYRK1A* (Fig. 5a and Supplementary Fig. S5). Intriguingly, all 14 of the remaining genes showed significantly higher expression in cDi21(M1 + M2) iPSCs than in cDi21(P + M2) (7 genes, Fig. 5b and Supplementary Fig. S6) or in both cDi21(P + M1) and cDi21(P + M2) iPSCs (7 genes, Fig. 5c), whereas no genes showed the reverse pattern. For most of these genes whose transcription was regulated in a chromosome-wide manner, the expression in cDi21(M1 + M2) iPSCs was upregulated to levels higher than that in disomy 21 iPSCs, although that in cDi21(P + M1) and cDi21(P + M2) lines remained similar. Taken together, these findings indicate that a group of genes on chromosome 21 underwent allele-specific transcriptional regulation in trisomy cells, and that interaction between nondisjunction-derived uniparental chromosomes may have caused this upregulation.

### Association of Transcriptional Regulation and Allelic Expression Pattern of Trisomic Genes

**Figure 6:**
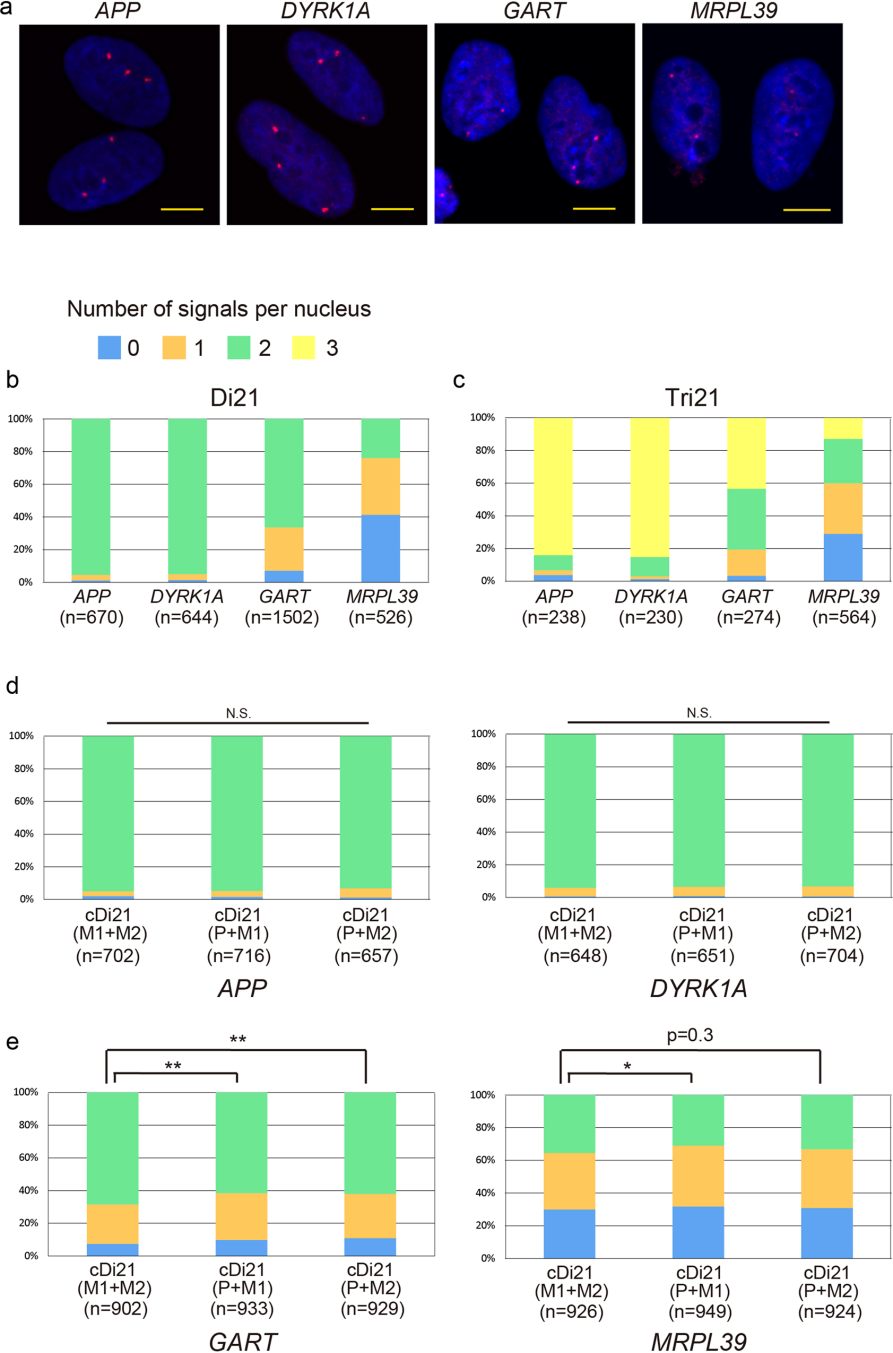
Transcriptional profiling of the genes on HSA21. (**a**) Nascent RNA FISH of each gene in Tri21 iPSCs. RNA was labelled with red probe. Nuclei were stained with DAPI. Scale bars represent 10 μm. (**b–e**) Number of RNA signals per nucleus in (**b**) Di21, (**c**) Tri21 and (**d,e**) cDi21 iPSCs. Error bars represent SEM. p values were determined by the chi-squared test. *p < 0.05, **p < 0.01.

Gene transcription is fundamentally infrequent and stochastic. The amplitude, duration, and frequency of transcriptional bursting varies significantly between individual genes. To characterise the transcriptionally regulated genes on chromosome 21, we performed nascent RNA FISH in various iPSC lines (Fig. 6a). Both *APP* and *DYRK1A*, whose mRNA expression levels were similar among the three corrected disomy 21 iPSC lines, showed a homogeneous pattern with biallelic expression in disomy 21 cells, and mainly triallelic expression in trisomy 21 iPSCs (Fig. 6b,c). In contrast, *GART* and *MRPL39*, whose gene expression levels were typically upregulated in maternal alleles, exhibited a more fluctuating pattern, which was accompanied by a substantial population of cells containing a single or no signal, in Di21 iPSCs. In Tri21 iPSCs, these tendencies were conserved, such that the expression pattern was more heterogeneous for *GART* and *MRPL39* genes (Fig. 6c). In corrected disomy 21 cells, signals of both *APP* and *DYRK1A* displayed patterns similar to that observed in Di21 iPSCs and there were no differences among cDi21(M1 + M2), -(P + M1) and -(P + M2) iPSC lines (Fig. 6d). *GART* and *MRPL39* again showed a fluctuating pattern of gene expression rather than an exclusively biallelic one (Fig. 6e). Notably, expression profiling of the *GART* gene in corrected disomy 21 cells indicated more cells with biallelic expression and fewer silenced cells in the maternal uniparental disomy (UPD) line, cDi21(M1 + M2) iPSCs, than in cDi21(P + M1) and cDi21(P + M2) iPSCs. The *MRPL39* gene also displayed a similar pattern. Taken together, these results indicate that two copies of the maternal chromosomes derived from meiotic nondisjunction persistently affect transcriptional regulation preferentially for transcriptionally suppressed and fluctuating genes.

## Discussion

Despite the general assumption that two homologous copies of each gene are expressed at similar levels in diploid mammalian cells, increasing evidence suggests that 0.5–24% of genes exhibit monoallelic expression^24–26^. Because the pathological phenotypes of DS are likely to be due to the extra copies of dosage-sensitive genes located in HSA21^13, 27^, the fluctuation in gene expression levels caused by allelic regulation can affect its phenotypic severity. However, the lack of a suitable model system in which gene expression from each allele can be precisely discriminated has hindered elucidation of the molecular mechanisms underlying DS pathology. In the present study, we successfully distinguished maternal chromosomes from paternal ones in the nucleus by applying 3D-FISH to genome-edited partial trisomy 21 iPSCs, in which regional deletion was specifically introduced to the paternal chromosome. To further evaluate the functional differences of each chromosome, we then developed a parent-of-origin-specific chromosome elimination technique in hiPSCs and established three corrected disomy 21 iPSC lines. These chromosome-edited disomy cells were confirmed to be functionally rescued regarding both proliferation and differentiation abilities, and enabled us to explore the individual effects of each parental chromosome on nuclear positioning and gene expression profile.

3D-FISH analysis of the Prtial-Tri21 iPSC line demonstrated that chromosome 21 exhibited nonrandom positioning depending on the parental origin, and that a pair of maternal chromosomes derived from meiotic nondisjunction formed an adjacent cluster at high frequency compared with the combination of the one paternal and one of the two maternal chromosomes. Several reports have suggested that homologous chromosomes are generally located far from each other and the distance between homologues is significantly greater than that between heterologues^28, 29^. Our findings indicated that, although the relative positioning of one paternal and one of the two copies of maternal chromosome 21 is normal, an extra copy of maternal chromosome 21 derived from meiotic nondisjunction is preferentially juxtaposed to its maternal homologue, leading to the distinctive intranuclear positioning pattern of trisomy 21.

The internal organisation of the CTs and the molecular mechanisms that underlie the formation of the trisomy-21-specific positioning pattern remain largely unknown. However, it has been observed that the location of chromosomes is linked to their gene density, with gene-rich chromosomes being located preferentially at the centre of the nucleus, whereas gene-poor ones accumulate at its periphery^30–34^. In addition, a previous study suggested that human chromosome 21 is constrained at the centre of the nucleus, possibly through interaction with the nucleoli^35^. Nucleolar organiser regions (NORs), potential anchoring sites to the nucleus^36^, are an important candidate for this constraint and the absence of these regions led to a rearrangement of the chromosome to a more peripheral position. Chromosome 21 is an acrocentric chromosome bearing NORs, and our observation that the supernumerary maternal chromosome 21 is located further from the nuclear membrane suggests that the existence of extra copies of NORs can actively perturb CTs through the effects of their anchoring to the nucleus. However, to clarify this issue, it will be necessary to analyse other aneuploidy-lacking NORs, such as in trisomy 18.

The contributions of each parental allele to transcription were almost identical in most trisomic genes, as expected. However, our data obtained using corrected disomy 21 iPSCs revealed a group of genes in which the expression levels of maternal alleles were upregulated more than for the paternal allele. Interestingly, we did not find exceptions to this expression pattern; that is, there were no genes in which expression was lower in the cDi21(M1 + M2) iPSC line than in cDi21(P + M1) or cDi21(P + M2) iPSCs. An RNA-FISH analysis indicated that this transcriptional upregulation in a pair of maternal alleles exclusively occurred in the genes that showed a more suppressed pattern of transcriptional bursting.

The functional mechanism underlying this chromosome-wide transcriptional regulation remains to be elucidated. One possibility is that distinctive localisation pattern and transcriptional upregulation observed in the pair of maternal chromosomes was exclusively affected by parent-of-origin effects. In euploid cells, maternal or paternal UPD of chromosome 21 has been reported not to be associated with imprinted pathological consequences^37, 38^. On the other hand, in trisomy 21 cells, there is a substantial evidence of maternally imprinted methylation marks at differentially methylated regions of the *WRB* gene^39^. Thus, there is still no conclusive proof that different parental origins of chromosome 21 affect the regulation of gene transcription, in turn contributing to the phenotypic variation. Our chromosome-edited cDi21(M1 + M2) iPSCs, which is an artificially generated maternal UPD cells, showed a dysregulation of transcription levels, and these results revealed, for the first time, that there are genes on chromosome 21 which are sensitive to maternal origin. On the other hand, 3D-FISH analysis of Partial-Mono21 iPSCs showed no significant differences in the distances to the nuclear membrane between maternal and paternal chromosomes 21, suggesting that there were no distinct effects of parental origin on the nuclear localisation pattern. Because approximately 5% of cases of trisomy 21 are derived from paternal nondisjunction^40^, a paternal-origin-dependent effect should be examined in future study.

Another possible explanation for this chromosome-wide transcriptional regulation is the effect of altered association with the nuclear membrane. A pair of maternal chromosomes, on which the expression levels were upregulated in some genes, were located relatively far from the nuclear membrane in human iPSCs. In addition to the general concept that small and gene-rich chromosomes are located in the centre of the nucleus, while large and gene-poor ones are preferentially localised near the nuclear periphery^9, 34, 41, 42^, chromosome positioning within the nucleus is correlated with the transcriptional status, and transcriptional activation of transgenes in human cells induced chromosome repositioning from the periphery toward the nuclear interior^43, 44^. Recent research has demonstrated that the genes positioned at the nuclear periphery tend to contain repressive histone marks, including H3K27me3 and H3K9me2, and interact with lamina-associated domains (LADs)^45, 46^. The relocation of the genes or chromosomes to the nuclear periphery resulted in suppression of the expression of some, but not all, genes^47, 48^. In addition, previous studies using foetal fibroblasts from a pair of monozygotic twins discordant for trisomy 21 demonstrated that the gene expression pattern was organised in domains, which correlate with LADs^49^. Although the dissociation of the genes from the lamina did not automatically result in gene activation, it is suggested that genes moving away from the lamina become unlocked for activation in the next differentiation step^32^. To clarify this, further epigenetic analysis including histone mark profiling and investigation of the association with LADs in trisomy 21 will be required in future study.

Another conceivable mechanism that may link the distinct positional pattern of trisomy 21 to the upregulated gene expression on the maternal alleles is interchromosomal interactions between homologous pairs. Homologous pairings are extensively observed in dipteran insects, such as *Drosophila*, even during interphase in the somatic nucleus of all cell types^50^. Such pairings cause physical interactions between elements on separate chromosomes and strongly affect gene transcription. Typically, the enhancer of one copy of a gene regulates the expression of its paired copy in *trans*, leading to either activation or inhibition of gene expression, called transvection^51, 52^. Although such chromosome-wide pairing is not generally observed in mammals, similar *trans* interaction in paired regions is known to affect the status of monoallelic expression or transcription level^53–55^. For example, in a previous study, abnormal somatic pairing of chromosome 19 caused a significant increase of gene expression in the paired region^56^. As mentioned above, homologous pairs of chromosomes are generally located in separate territories in human and rarely interact with each other^35, 57, 58^. There may be an active mechanism of separation of homologous chromosomes to avoid disruption of the gene expression balance^59^. However, the presence of supernumerary chromosomes in aneuploidy may perturb this coordinated status by raising the frequency of inadequate chromosomal pairing, which can induce the coregulation of both alleles of monoallelically expressed genes, leading to the transcriptional upregulation. Our observations, at least in part, are consistent with these hypotheses.

The pathological significance of these elevated gene activities in maternal chromosomes remains unclear. Detailed analysis of the gene expression profile in differentiated cells will be required to elucidate the mechanism underlying DS and develop new therapies for this condition. Our chromosome-editing technologies and collection of DS-derived iPSC lines should provide a useful resource to study human diseases associated with aneuploidy.

## Methods

### Generation and Culture of hiPSCs

Human iPSCs (hiPSCs) were established and cultured as previously described^17^. In brief, iPSCs were generated from either cord blood cells, or dermal fibroblast, a kind gift from Dr. Toshiyuki Yamamoto (Tokyo Women’s Medical University Institute for Integrated Medical Science, Tokyo, Japan), using a Sendaivirus vector encoding tetracistronic factors (*OCT3/4, SOX2, KLF4 and c-Myc*) and maintained on mitomycin C (Sigma)-inactivated mouse embryonic fibroblasts in hES medium containing DMEM/F12 (Wako), 20% KnockOut Serum Replacement (Gibco), 2 mM L-alanyl-L-glutamine (Wako), 1% MEM nonessential amino acid solution (Wako), 0.1 mM 2-mercaptoethanol (Sigma) and 5 ng/mL basic fibroblast growth factor (Katayama Chemical). Cultures were passaged every 6–8 days. This study was approved by the Ethics Committee of Osaka University Graduate School of Medicine (approval no. 13123-823). Informed consent was obtained from each patient’s guardians in accordance with the Declaration of Helsinki. All experiments were performed in accordance with the approved guidelines.

### Teratoma Formation

A total of 0.5–1.0 × 10^6^ Tri21 iPSCs were injected into the testes of mice with severe combined immunodeficiency. After about 12 weeks mice were perfusion fixed and then the teratomas that formed as a result were excised and analysed histologically using H&E staining. All protocols used for animal experiments in this study were approved by the Animal Experimentation Committee of Osaka University (approval no. 27-090-002) and performed in accordance with the approved guidelines.

### Haematopoietic and Neural Cell Differentiation

Human iPSCs were differentiated into haematopoietic cells as described previously^17^. Neural cell differentiation was performed in accordance with previous protocols^60^ with slight modifications. iPSC colonies were mechanically dissociated into small aggregate suspensions and then plated onto Costar six-well ultra-low-attachment plates (Corning, NY, USA) in DFK medium (hES medium with 10 μM SB431542, 2 μM Compound C and 1% penicillin/streptomycin) with 10 μM ROCK inhibitor (Y27632; Reagents Direct). Medium without ROCK inhibitor was changed on day 2, day 4, and day 6. On day 8, EBs were collected and plated on Matrigel-coated 12-well plates in DFN2D medium containing DMEM/F12, 50% Neurobasal medium, 0.5% N2 supplement, 0.5% B27 supplement, 1% L-alanyl-L-glutamine, 1% MEM nonessential amino acid solution, 1% glutamax-1,2-mercaptethanol, 1% penicillin/streptomycin and 20 ng/µl basic fibroblast growth factor. Medium was changed every 2 days for 6 days. On day 18, cells were dissociated using TryPLE Express (Life Technologies) and plated on 24-well plates. Passage was performed every 2 days. Immunohistochemistry was performed using the following primary antibodies: anti-Sox1 (R&D Systems) and anti-Nestin (eBiosciences).

### Establishment of Corrected Disomy 21 iPSC Lines

Chromosome elimination was performed as previously described with some modification for application on hiPSCs^21^. The chromosome elimination cassette was designed to bear positive/negative drug selection marker (puroΔTK) between oppositely oriented loxP sites. This chromosome elimination cassette was introduced using the TALENs into the single locus of *RUNX1* or *ETS2* in Tri21 iPSCs. The construction of TALENs, TALEN-mediated gene targeting in hiPSCs and genotyping using STR and SNP markers were performed as previously described^17^. At 1 day prior to transfection (day −1), hiPSC colonies were dissociated into single cells using TrypLE Express (Life Technologies) with 10 μM ROCK inhibitor (Y27632; Reagents Direct, Encinitas, CA, USA). On the day of transfection (day 0), 0.5–1.0 × 10^6^ cells were dissociated with TrypLE Express, mixed with TALENs (left 1 μg + right 1 μg) and chromosome elimination cassette (5 μg), and then electroporated using Neon Transfection System (Life Technologies). Electroporated cells were plated onto 10-cm dish with DR-4 IRR MEFs (GlobalStem, Rockville, MD, USA). On day 4, drug selection with puromycin (0.5–1.0 μg/mL) was initiated and the resulting colonies were selected on days 13–16. PCR-positive clones were expanded further, and homologous recombination was verified by Southern blot analysis. The parental origin of the targeted allele was analysed by STR or SNP genotyping. The resultant targeted clones were subjected to Cre recombinase-mediated chromosome elimination followed by FIAU negative selection. Chromosome elimination was verified using STR and karyotype analysis.

### DNA and Nascent RNA FISH

DNA FISH was carried out as previously described^17, 61^. In nascent RNA FISH, cellular DNA was not denatured and hybridisation was performed with 4 U/µl RNase OUT (Invitrogen) to avoid the elimination of RNA. *APP* and *DYRK1A* were probed with BACs from BACPAC Resources (*APP*, RP11-910G8; *DYRK1A*, Rp11-105O24), while *GART* and *MRPL39* were probed with PCR products (39 Kb and 22 Kb, respectively). Primer sequences are provided in Supplementary Table S1. DNA probes were labelled by nick translation with either digoxigenin-16-dUTP or biotin-11-dUTP (Roche). Both digoxigenin- and biotin-labelled probes were used for DNA FISH, while digoxigenin-labelled probe was only used for RNA FISH. In DNA FISH, images were captured using a Carl Zeiss LSM 710 and processed using Volocity software (PerkinElmer). In RNA FISH, images were acquired and processed using IN Cell Analyzer 6000 (GE Healthcare). The numbers of signals were counted blindly.

### Cell Preparation for 3D-FISH

Human iPSC colonies were dissociated into single cells and cultured on coverslips (24 × 60 mm, Matsunami) for a few days. Cells on the coverslips were washed twice with phosphate-buffered saline (PBS) for 3 min, fixed in 4% paraformaldehyde (PFA) in 0.3 × PBS for 10 min and washed again in PBS. For permeabilisation, cells were treated with 0.5% saponin (Nacalai Tesque) and 0.5% Triton X-100 in PBS for 20 min, incubated in 20% glycerol (Merck Millipore) in PBS for at least 30 min and subjected to repeated freeze–thaw cycles in liquid nitrogen five times. After washing in PBS for 2 min, cells were incubated in 0.1 N HCl for 10 min, washed in PBS for 5 min, treated in 0.02% pepsin in 0.01 N HCl at 37 °C for 4 min and washed with 0.05 M MgCl_2_ in PBS for 5 min. Next, cells were postfixed with 1% PFA in PBS for 10 min, and washed in PBS for 5 min and then in 2 × SSC for 5 min. Cells on coverslips were stored at 4 °C in 50% formamide (Wako) in 2 × SSC until hybridisation.

### 3D-FISH Analysis

For the 3D-FISH analysis, digoxigenin-labelled *DYRK1A* probe was used, as described above in the section on DNA and RNA FISH. Human chromosome 21 long arm-specific painting probe was kindly provided by Prof. Dr. Thomas Cremer (LMU, Munich, Germany), and labelled with dinitrophenyl (DNP) as previously described^62, 63^. A total of 2 μg of each labelled probe and 3 μg of Cot-1 DNA were mixed and subjected to ethanol precipitation, and then resuspended in 3 µl of hybridisation solution (50% formamide and 10% dextran sulphate in 2 × SSC). Probes were predenatured at 80 °C for 6 min and placed on ice for 1 min. The denatured probes were then applied to fixed cells on the coverslip, covered with coverslips (18 × 18 mm, Matsunami) and sealed with Fixogum Rubber Cement. The cells were then denatured at 75 °C for 4 min and hybridisation was performed in a moist chamber at 37 °C for 3 days. After hybridisation, the cells were washed in 2 × SSC twice, 0.1 × SSC at 62.5 °C for 5 min three times and 4 × SSC for 2 min. The cells were then blocked in 4 × SSC containing 5% BSA and 0.2% Tween-20 for 30 min at 37 °C prior to probe detection. The probe detection was performed in detection buffer (4 × SSC containing 5% BSA and 0.2% Tween-20) with a rabbit anti-DNP antibody (Sigma) and a mouse anti-digoxigenin antibody (Sigma) for 50 min, followed by amplification with a goat anti-rabbit, Alexa 488-conjugated antibody (Invitrogen/Molecular Probes) and a sheep anti-mouse, Cy3-conjugated antibody (Dianova/Jackson ImmunoResearch Lab) for 50 min. The cells were washed after each step (three times in 4 × SSC containing 0.2% Tween-20 for 5 min). The cells were then washed again in 4 × SSC and MilliQ water. Nuclear DNA was counterstained with DAPI and the slides were mounted in Vectashield Antifade (Vector). Images were captured using a Carl Zeiss LSM 710 and processed using Volocity software (PerkinElmer, Waltham, MA, USA).

### 3D Distance Measurements in 3D-FISH

The measurements and 3D reconstructions were performed using Imaris and ImarisCell software (Bitplane). Nuclear volume, position of the fluorescence peak centre (FPC) of each signal, distance from the nuclear centre to FPC and distance from FPC to the nearest nuclear membrane were determined. Distance between two FPCs and interior angles at the vertex were also calculated as described previously^62^.

### EdU Assay

One day prior to the EdU treatment, hiPSC colonies were dissociated into single cells using TrypLE Express (Life Technologies) with 10 μM ROCK inhibitor (Y27632; Reagents Direct, Encinitas, CA, USA) and 2 × 10^4^ cells were plated onto a Matrigel-coated 12-well plate. On the day of EdU treatment, cells were cultured with 10 μM EdU for 1.5 h and EdU-positive cells were detected using Click-iT EdU Alexa Fluor 488 Flow Cytometry kit (Life Technologies).

### Quantitative RT-PCR

Human iPSC colonies were dissociated into single cells using TrypLE Express with 10 μM ROCK inhibitor and 2 × 10^5^ cells were plated in a T25 flask. The medium of the cells was supplemented with 10 μM ROCK inhibitor 2 days later. After at least 8 h, cells were collected and RNA was extracted using NucleoSpin RNA Kit (TaKaRa). Reverse transcription was performed using a ReverTra Ace qPCR RT Kit (TOYOBO) and quantitative PCR was performed using THUNDERBIRD SYBR qPCR Mix (TOYOBO). Gene expression was calculated relative to that of β-actin (ACTB). All primer sequences are provided in Supplementary Table S2.

### Statistical Analysis

Student’s t-tests for normally distributed variables and Mann–Whitney U tests for non-normally distributed variables were used to compare the means of two groups. Dunnett’s or Steel–Dwass tests were used to compare the means of three or more groups. In EdU assays, Student’s t-tests were used to analyse the proportion of EdU-positive cells in each hiPSC line. In RNA FISH, the chi-squared test was used to assess the proportion of biallelic expression in each cDi21 iPSC line. A value of p < 0.05 was considered significant. Values are reported as mean ± SEM.

## Electronic supplementary material

Supplementary information

## References

[1] Kouzarides T (2007). Chromatin modifications and their function. Cell.

[2] Fuks F (2005). DNA methylation and histone modifications: teaming up to silence genes. Curr Opin Genet Dev.

[3] Nguyen HQ, Bosco G (2015). Gene Positioning Effects on Expression in Eukaryotes. Annu Rev Genet.

[4] Lanctot C, Cheutin T, Cremer M, Cavalli G, Cremer T (2007). Dynamic genome architecture in the nuclear space: regulation of gene expression in three dimensions. Nat Rev Genet.

[5] Cremer T, Cremer M (2010). Chromosome territories. Cold Spring Harb Perspect Biol.

[6] Cremer T (2015). The 4D nucleome: Evidence for a dynamic nuclear landscape based on co-aligned active and inactive nuclear compartments. FEBS Lett.

[7] Parada LA, McQueen PG, Misteli T (2004). Tissue-specific spatial organization of genomes. Genome Biol.

[8] Mayer R (2005). Common themes and cell type specific variations of higher order chromatin arrangements in the mouse. BMC Cell Biol.

[9] Tanabe H (2002). Evolutionary conservation of chromosome territory arrangements in cell nuclei from higher primates. Proc Natl Acad Sci USA.

[10] Fraser P, Bickmore W (2007). Nuclear organization of the genome and the potential for gene regulation. Nature.

[11] Marella NV, Bhattacharya S, Mukherjee L, Xu J, Berezney R (2009). Cell type specific chromosome territory organization in the interphase nucleus of normal and cancer cells. J Cell Physiol.

[12] Qureshi IA, Mehler MF (2010). Impact of nuclear organization and dynamics on epigenetic regulation in the central nervous system: implications for neurological disease states. Ann N Y Acad Sci.

[13] Megarbane A (2009). The 50th anniversary of the discovery of trisomy 21: the past, present, and future of research and treatment of Down syndrome. Genet Med.

[14] Dierssen M, Herault Y, Estivill X (2009). Aneuploidy: from a physiological mechanism of variance to Down syndrome. Physiol Rev.

[15] Steingass KJ, Chicoine B, McGuire D, Roizen NJ (2011). Developmental disabilities grown up: Down syndrome. J Dev Behav Pediatr.

[16] Kahlem P (2004). Transcript level alterations reflect gene dosage effects across multiple tissues in a mouse model of down syndrome. Genome Res.

[17] Banno K (2016). Systematic Cellular Disease Models Reveal Synergistic Interaction of Trisomy 21 and GATA1 Mutations in Hematopoietic Abnormalities. Cell Rep.

[18] Lockstone HE (2007). Gene expression profiling in the adult Down syndrome brain. Genomics.

[19] Antonarakis SE, Lyle R, Dermitzakis ET, Reymond A, Deutsch S (2004). Chromosome 21 and down syndrome: from genomics to pathophysiology. Nat Rev Genet.

[20] Paz N (2013). Combined fluorescent-chromogenic *in situ* hybridization for identification and laser microdissection of interphase chromosomes. PLoS One.

[21] Matsumura H (2007). Targeted chromosome elimination from ES-somatic hybrid cells. Nat Methods.

[22] Jiang J (2013). Translating dosage compensation to trisomy 21. Nature.

[23] Yamamoto T (2011). Clinical manifestations of the deletion of Down syndrome critical region including DYRK1A and KCNJ6. Am J Med Genet A.

[24] Eckersley-Maslin MA, Spector DL (2014). Random monoallelic expression: regulating gene expression one allele at a time. Trends Genet.

[25] Deng Q, Ramskold D, Reinius B, Sandberg R (2014). Single-cell RNA-seq reveals dynamic, random monoallelic gene expression in mammalian cells. Science.

[26] Reinius B, Sandberg R (2015). Random monoallelic expression of autosomal genes: stochastic transcription and allele-level regulation. Nat Rev Genet.

[27] Kahlem P (2006). Gene-dosage effect on chromosome 21 transcriptome in trisomy 21: implication in Down syndrome cognitive disorders. Behav Genet.

[28] Brianna Caddle L (2007). Chromosome neighborhood composition determines translocation outcomes after exposure to high-dose radiation in primary cells. Chromosome Res.

[29] Khalil A (2007). Chromosome territories have a highly nonspherical morphology and nonrandom positioning. Chromosome Res.

[30] Boyle S (2001). The spatial organization of human chromosomes within the nuclei of normal and emerin-mutant cells. Hum Mol Genet.

[31] Bridger JM, Boyle S, Kill IR, Bickmore WA (2000). Re-modelling of nuclear architecture in quiescent and senescent human fibroblasts. Curr Biol.

[32] Cremer T, Cremer C (2001). Chromosome territories, nuclear architecture and gene regulation in mammalian cells. Nat Rev Genet.

[33] Cremer M (2001). Non-random radial higher-order chromatin arrangements in nuclei of diploid human cells. Chromosome Res.

[34] Tanabe H, Habermann FA, Solovei I, Cremer M, Cremer T (2002). Non-random radial arrangements of interphase chromosome territories: evolutionary considerations and functional implications. Mutat Res.

[35] Heride C (2010). Distance between homologous chromosomes results from chromosome positioning constraints. J Cell Sci.

[36] Hernandez-Verdun D (2006). The nucleolus: a model for the organization of nuclear functions. Histochem Cell Biol.

[37] Blouin JL, Avramopoulos D, Pangalos C, Antonarakis SE (1993). Normal phenotype with paternal uniparental isodisomy for chromosome 21. Am J Hum Genet.

[38] Rogan PK, Sabol DW, Punnett HH (1999). Maternal uniparental disomy of chromosome 21 in a normal child. Am J Med Genet.

[39] Alves da Silva AF (2016). Trisomy 21 Alters DNA Methylation in Parent-of-Origin-Dependent and -Independent Manners. PLoS One.

[40] Petersen MB (1993). Paternal nondisjunction in trisomy 21: excess of male patients. Hum Mol Genet.

[41] Scheuermann MO (2004). Topology of genes and nontranscribed sequences in human interphase nuclei. Exp Cell Res.

[42] Croft JA (1999). Differences in the localization and morphology of chromosomes in the human nucleus. J Cell Biol.

[43] Tumbar T, Belmont AS (2001). Interphase movements of a DNA chromosome region modulated by VP16 transcriptional activator. Nat Cell Biol.

[44] Chuang CH (2006). Long-range directional movement of an interphase chromosome site. Curr Biol.

[45] Guelen L (2008). Domain organization of human chromosomes revealed by mapping of nuclear lamina interactions. Nature.

[46] Pickersgill H (2006). Characterization of the Drosophila melanogaster genome at the nuclear lamina. Nat Genet.

[47] Reddy KL, Zullo JM, Bertolino E, Singh H (2008). Transcriptional repression mediated by repositioning of genes to the nuclear lamina. Nature.

[48] Finlan LE (2008). Recruitment to the nuclear periphery can alter expression of genes in human cells. PLoS Genet.

[49] Letourneau A (2014). Domains of genome-wide gene expression dysregulation in Down’s syndrome. Nature.

[50] McKee BD (2004). Homologous pairing and chromosome dynamics in meiosis and mitosis. Biochim Biophys Acta.

[51] Duncan IW (2002). Transvection effects in Drosophila. Annu Rev Genet.

[52] Ou SA (2009). Effects of chromosomal rearrangements on transvection at the yellow gene of Drosophila melanogaster. Genetics.

[53] Hewitt SL (2009). RAG-1 and ATM coordinate monoallelic recombination and nuclear positioning of immunoglobulin loci. Nat Immunol.

[54] Krueger C (2012). Pairing of homologous regions in the mouse genome is associated with transcription but not imprinting status. PLoS One.

[55] Schoenfelder S, Clay I, Fraser P (2010). The transcriptional interactome: gene expression in 3D. Curr Opin Genet Dev.

[56] Koeman JM (2008). Somatic pairing of chromosome 19 in renal oncocytoma is associated with deregulated EGLN2-mediated [corrected] oxygen-sensing response. PLoS Genet.

[57] Selvaraj SJRD, Bansal V, Ren B (2013). Whole-genome haplotype reconstruction using proximity-ligation and shotgun sequencing. Nat Biotechnol.

[58] Rao SS (2014). A 3D map of the human genome at kilobase resolution reveals principles of chromatin looping. Cell.

[59] Joyce EF, Erceg J, Wu CT (2016). Pairing and anti-pairing: a balancing act in the diploid genome. Curr Opin Genet Dev.

[60] Kim DS (2012). Highly pure and expandable PSA-NCAM-positive neural precursors from human ESC and iPSC-derived neural rosettes. PLoS One.

[61] Gimelbrant A, Hutchinson JN, Thompson BR, Chess A (2007). Widespread monoallelic expression on human autosomes. Science.

[62] Kawamura R (2012). Visualization of the spatial positioning of the SNRPN, UBE3A, and GABRB3 genes in the normal human nucleus by three-color 3D fluorescence *in situ* hybridization. Chromosome Research.

[63] Maejima T (2014). Direct evidence for pitavastatin induced chromatin structure change in the KLF4 gene in endothelial cells. PLoS One.

