# Supplementary material for: A Pair of Maternal Chromosomes Derived from Meiotic Nondisjunction in Trisomy 21 Affects Nuclear Architecture and Transcriptional Regulation
Source: Sci Rep. 2017 Apr 10;7:764. doi: 10.1038/s41598-017-00714-7 (PMC5429678; doi:10.1038/s41598-017-00714-7)
Supplement: Supplementary file 1 — Supplementary information [file 41598_2017_714_MOESM1_ESM.pdf]

Supplementary Information

A Pair of Maternal Chromosomes Derived from Meiotic Nondisjunction in Trisomy 21 Affects Nuclear Architecture and Transcriptional Regulation

Sayaka Omori,[1,2] Hideyuki Tanabe,[3] Kimihiko Banno,[1] Ayumi Tsuji,[1] Nobutoshi Nawa,[1] Katsuya Hirata,[1] Keiji Kawatani,[1] Chikara Kokubu,[4] Junji Takeda,[4] Hidetoshi Taniguchi,[1] Hitomi Arahori,[1] Kazuko Wada,[1] Yasuji Kitabatake,[1,*] and Keiichi Ozono[1]

[1]Department of Pediatrics, Graduate School of Medicine, Osaka University, Suita, Osaka 565-0871, Japan

[2]Japan Society for the Promotion of Science, Chiyoda-ku, Tokyo 102-0083, Japan

[3]Department of Evolutionary Studies of Biosystems, School of Advanced Sciences, SOKENDAI (The Graduate University for Advanced Studies), Shonan Village, Hayama, Kanagawa 240-0193, Japan

[4]Department of Genome Biology, Graduate School of Medicine, Osaka University, Suita, Osaka 565-0871, Japan

*Correspondence:

Yasuji Kitabatake, Department of Pediatrics, Graduate School of Medicine, Osaka University, 2-2 Yamadaoka, Suita, Osaka 565-0871, Japan

Tel: +81-6-6879-3932; Fax: +81-6-6879-3939; E-mail: ykitaba@ped.med.osaka-u.ac.jp

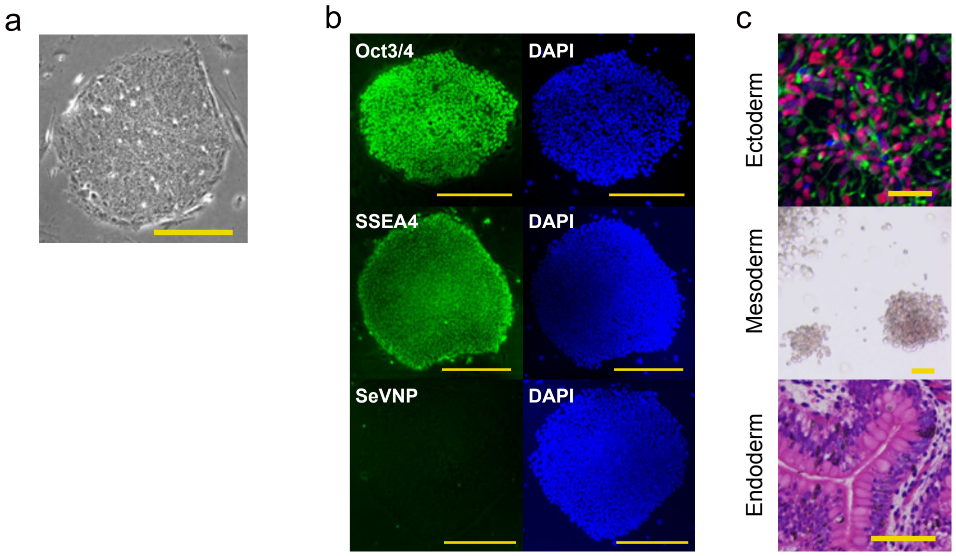

Supplementary Figure S1. Generation of Tri21 iPSCs.

(a) Photomicrograph of Tri21 iPSCs derived from mononuclear cells from a Down syndrome male. Scale bars represent 500µm.

(b) Immunocytochemistry for OCT3/4, SSEA4 and SeVNP in Tri21 iPSCs. Nuclei were stained with DAPI. Scale bars represent 500µm.

(c) Tri21 iPSCs represented differentiation into three germ layers: ectoderm (neural cells, immunohistochemistry of Sox1 (red) and Nestin (green)), mesoderm (hematopoietic colonies) and endoderm (teratoma formation, H&E staining). Scale bars represent 50µm.

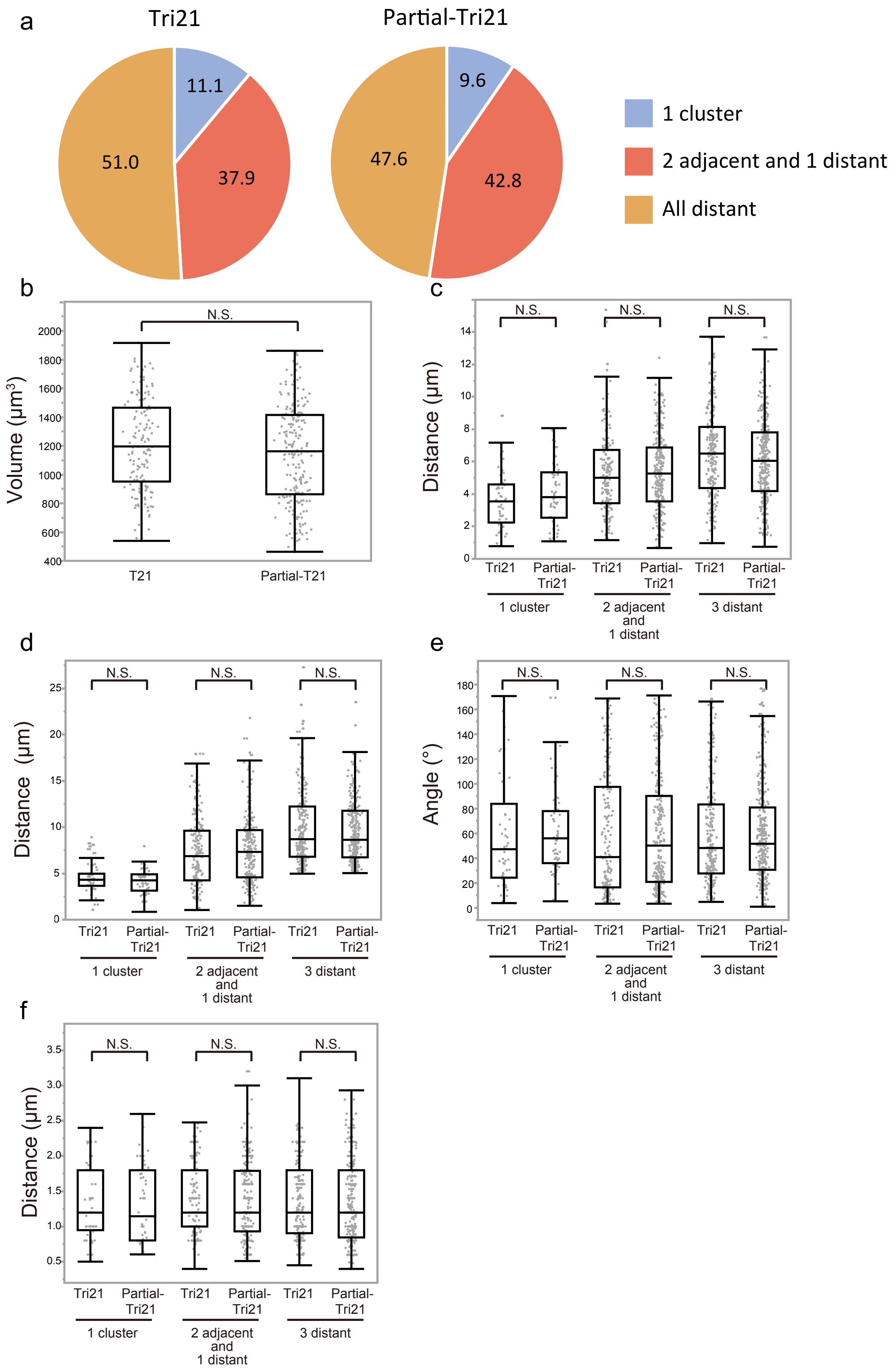

Supplementary Figure S2. Chromosome positioning of chromosome 21 compared Tri21 with Partial-Tri21 iPSCs

(a) Percentage of three localization patterns of chromosome 21 in Tri21 (n=153) and Partial-Tri21 (n=208) iPSCs.

(b–f) Chromosomal localization of chromosome 21 in Tri21 and Partial-Tri21 iPSCs. Three localization patterns of chromosome 21: 1 cluster (Tri21, n=17; Partial-Tri21, n=20), 2 adjacent and 1 distant (Tri21, n=58; Partial-Tri21, n=80) and all distant (Tri21, n= 78; Partial-Tri21, n=99). Measurements including (b) the nuclear volume, (c) the distance from the nuclear center to each chromosome 21, (d) the distance between two chromosomes 21, (e) interior angles at the vertex and (f) the distance from chromosome 21 to the nearest nuclear membrane.

The term "adjacent" here refers to the distance between copies of chromosome 21 being under 5 µm. Box plot represents 25th–75th percentile range ± min–max. p values were determined by the Mann-Whitney U test.

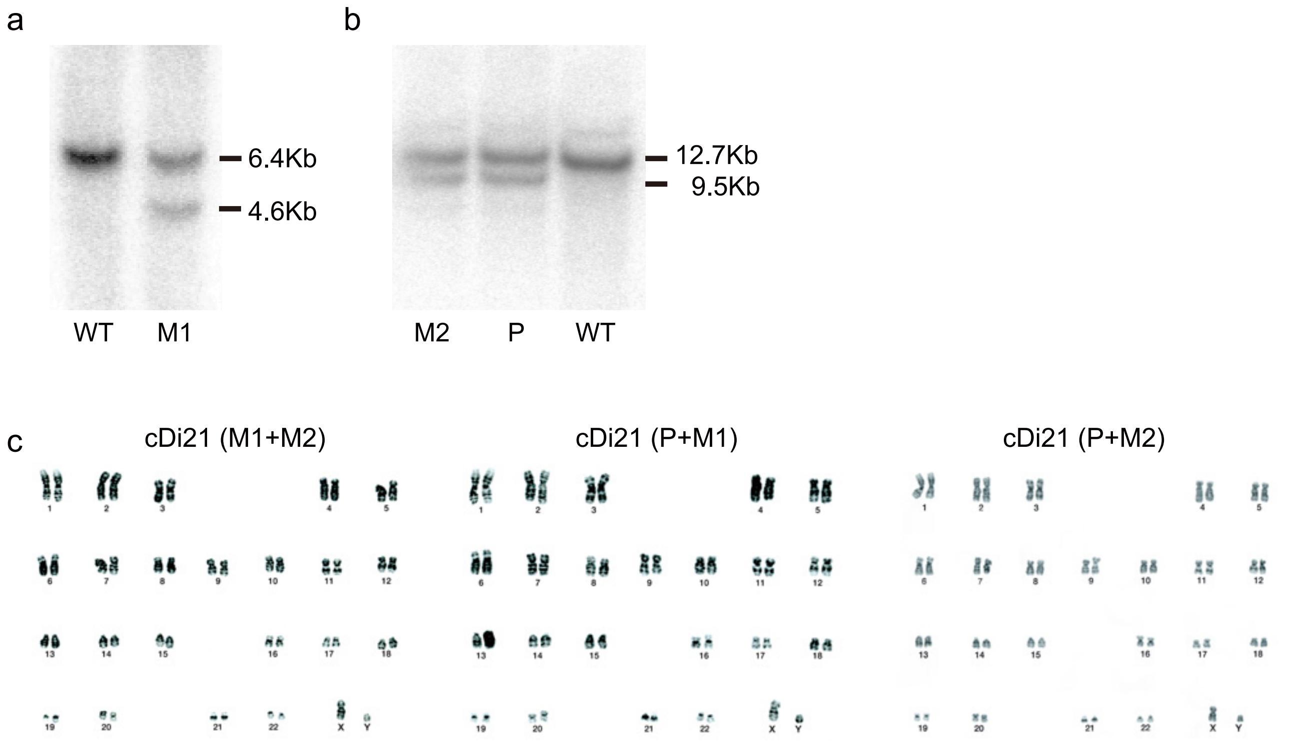

Supplementary Figure S3. Southern blot and karyotype analysis in targeted chromosome elimination

(a and b) Southern blot analysis of iPSC lines before (WT) and after (P, M1 and M2) targeting using 3’ probes in (a) *RUNX1* (probe; Figure 1b) and (b) *ETS2* (probe; Figure 1c) locus. P, M1 and M2 indicate targeted allele in *RUNX1* (M1) and *ETS2* (P or M2). Parental origin of targeted allele was analyzed by STR or SNP genotyping. Expected fragment sizes: *RUNX1* probe (NcoI digest) wild-type allele, 4.6 kb; targeted allele, 6.4 kb. *ETS2* Probe (SpeI digest) wild-type allele, 9.5 Kb; targeted allele, 12.7 kb.

(c) Karyotype analysis in each cDi21 iPSC line.

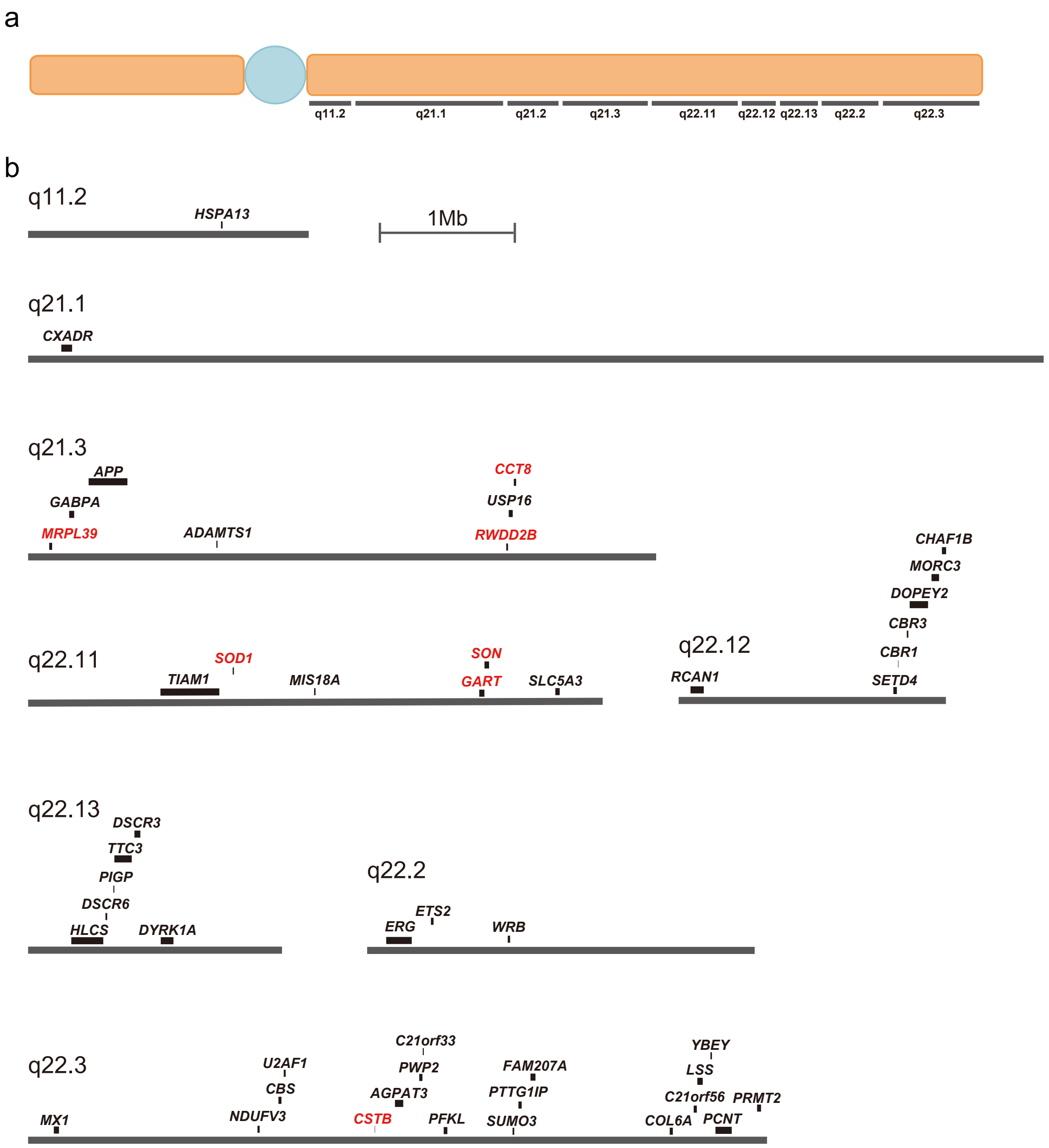

Supplementary Figure S4. Gene positions on long arm of chromosome 21

(a) Chromosome regions of long arm of chromosome 21.

(b) Gene location in each region of chromosome 21. Black boxes indicate gene bodies. Genes in Figure 4c are shown in red.

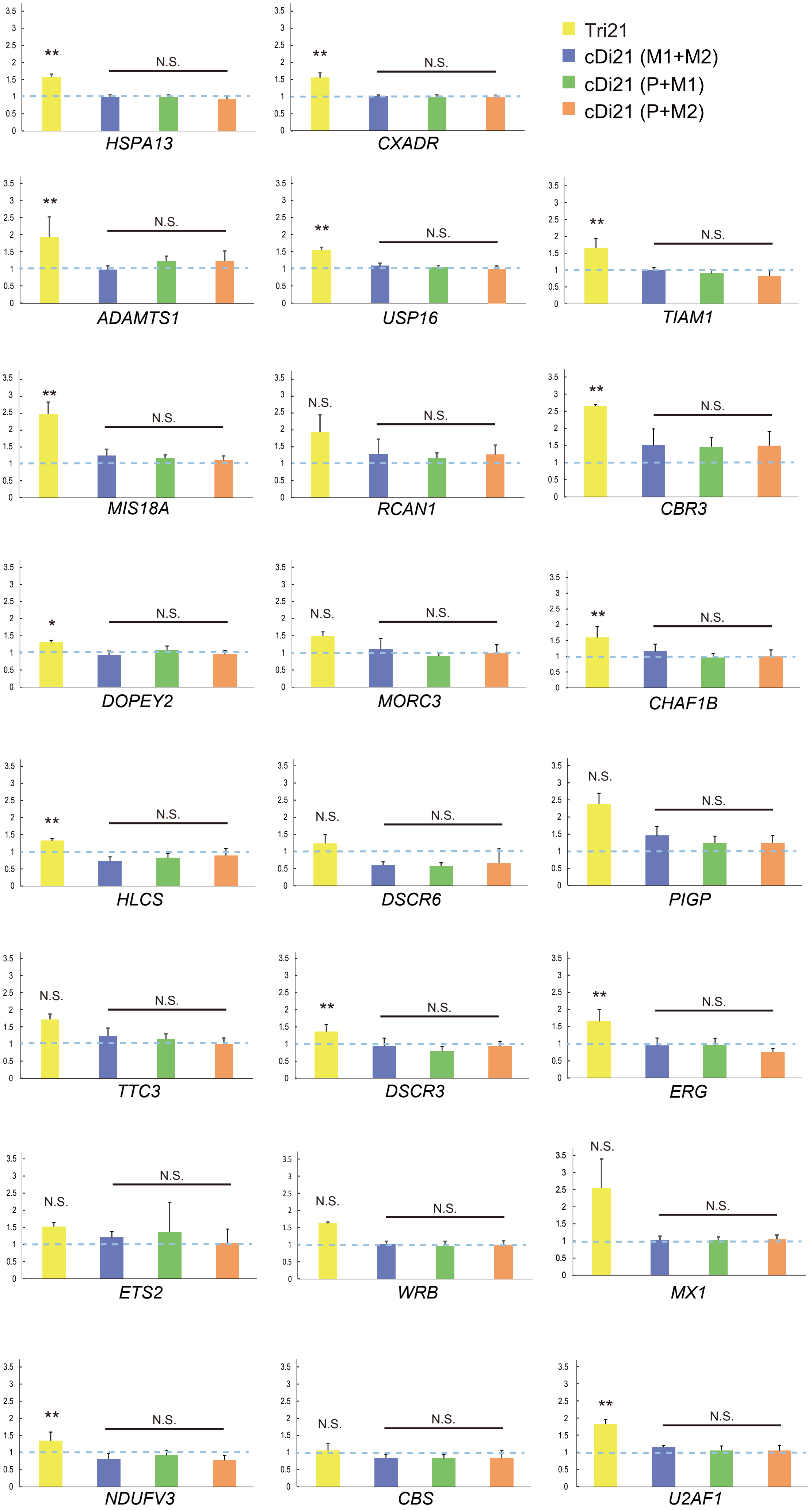

Tri21
cDi21 (M1+M2)
cDi21 (P+M1)
cDi21 (P+M2)
HSPA13
CXADR
ADAMTS1
USP16
TIAM1
MIS18A
RCAN1
CBR3
DOPEY2
MORC3
CHAF1B
HLCS
DSCR6
PIGP
TTC3
DSCR3
ERG
ETS2
WRB
MX1
NDUFV3
CBS
U2AF1
N.S.
**
*

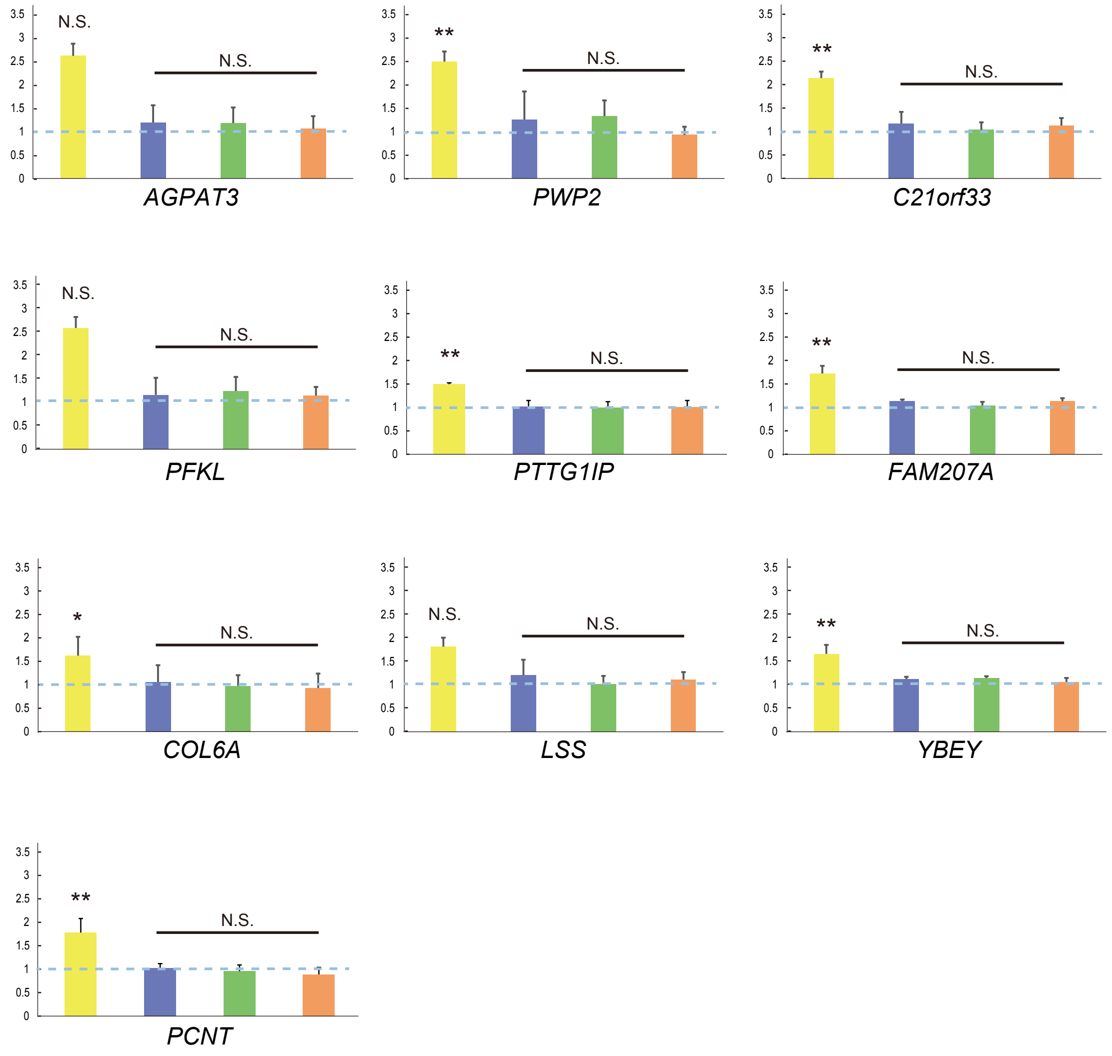

Supplementary Figure S5. Genes whose expression levels were not much different between cDi21 iPSCs

Relative expression levels of genes on chromosome 21 in Tri21 (yellow, n=3), cDi21 (M1+M2) (blue, n=9), cDi21 (P+M1) (green, n=9) and cDi21 (P+M2) (orange, n=9) iPSCs. Gene expression was normalized to that of Di21.

Error bars represent SEM. p values were determined by the Student' s t test or Mann-Whitney U test. *$p < 0.05$, **$p < 0.01$.

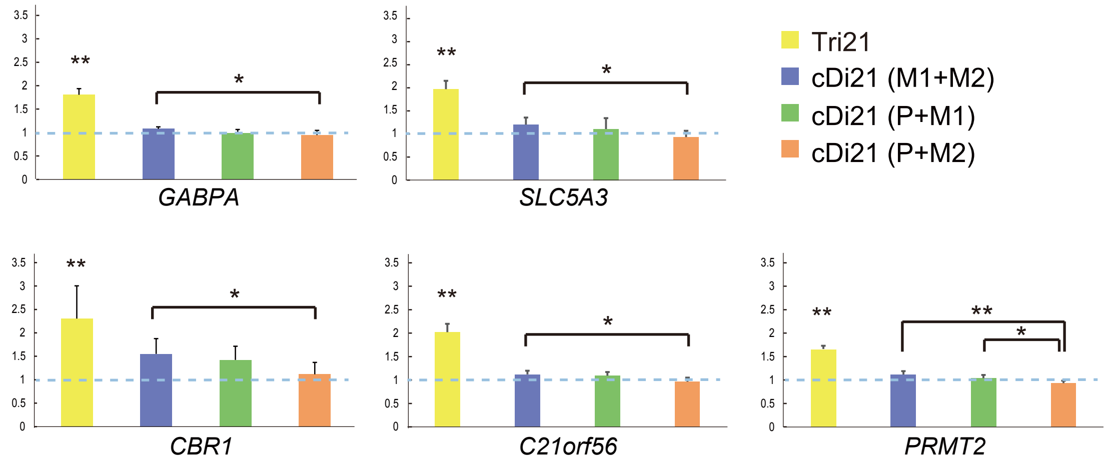

Supplementary Figure S6. Genes whose expression levels were higher in cDi21 (M1+M2) than in cDi21 (P+M2)

Relative expression levels of genes on chromosome21 in Tri21 (yellow, n=3), cDi21 (M1+M2) (blue, n=9), cDi21 (P+M1) (green, n=9) and cDi21 (P+M2) (orange, n=9) iPSCs. Gene expression was normalized to that of Di21.

Error bars represent SEM. p values were determined by the Student's t test or Mann-Whitney U test. *$p < 0.05$, **$p < 0.01$.

Supplementary Table S1. Primers for RNA FISH

Gene	No.	Forward primer (5'-3')	Reverse primer (5'-3')
GART	1	CCCTTGTTTATACAGGAAGC	GTAATGGCGCCTGCGTCAGA
	2	TCTGACGCAGGCGCCATTAC	GCATCAGCTTTGGGGCCATT
	3	AATGGCCCCAAAGCTGATGC	AATCCAAGACCACGACAGGC
	4	GCCTGTCGTGGTCTTGGATT	CATGAGAACTTGGGCGAGAG
	5	CTCTCGCCCAAGTTCTCATG	ATTGTGCCACTGCACTCCAG
	6	CTGGAGTGCAGTGGCACAAT	GGAATCTACGATTATGGGATC
	7	GATCCCATAATCGTAGATTCC	GTCTGGAACTCCTGACATCAGG
	8	CCTGATGTCAGGAGTTCCAGAC	TTCAGCTTCCAGAGTAGCTG
MRPL39	1	CCAGTGATTTGCATTCCTCC	AAATGCCAGAGTGAAGGAAG
	2	TAATCTTCCTTCACTCTGGC	TGTATATACAGGGACCTCAC
	3	GTTGCATACAGTGAGGTCCC	AGAAACCACAAGTGTTCCTG
	4	TCAGGAACACTTGTGGTTTC	CCTAAGTAAAAGAGGCACAC
	5	GTGTGCCTCTTTTACTTAGG	GCACAAGGATATTTTCTGCG

Supplementary Table S2. Primers for Quantitative RT-PCR

Gene	Forward primer (5'-3')	Reverse primer (5'-3')
ACTB	TCAAGATCATTGCTCCTCCTGAG	ACATCTGCTGGAAGGTGGACA
HSPA13	CCAGAGAGATGACGATCTTAGGA	TCACTTTAGGAGTAGGCAATGGT
CXADR	GTGCTCCTGTGCGGAGTAG	ATGGCAGATAGGCAGTTTCCC
MRPL39	CCGACAGAATTGACAGAAATGCG	CTTCTCAGTTCGGGGAGTTAATG
GABPA	AAAGAGCGCCGAGGATTTCAG	CCAAGAAATGCAGTCTCGAG
APP	CTTGGAGAGGTGTGCTCTGAA	GGTTCCTGGGTAGTCTTGAGT
ADAMTS1	CAGAGCACTATGACACAGCAA	AGCCATCCCAAGAGTATCACA
RWDD2B	AAATGGCGATGTTTTCTCTGGC	GGCGTGTTCTCTAACCCACTC
USP16	AGCCTTCAGTTTGGCTGTGT	GGCTTTGGAGTTGTAATGCTGG
CCT8	TTTTTCCTGATTCCGGCCATT	AACCATGCCATGCAATACTGA
TIAM1	ATGACGCTACATATTTGGCTGAG	ACCCAAGATTTCTTCGTTGCTT
SOD1	GGTGGGCCAAAGGATGAAGAG	CCACAAGCCAAACGACTTCC
MIS18A	AGCTCCATGAGCGAAGACG	ACGTTTGGATAGCTTCTGTTCC
GART	CTGGGATTGTTGGGAACCTGAG	ACCAAAGCAGGGAAGTCTGCAC
SON	TTTCTGGAGCCCTCTTTCAA	GCCAGGAGTCTACCAAATGAAG
SLC5A3	TCTTGGAGCAGTCCGTTTGA	GATGAAGCCCGGCCTATTATC
RCAN1	GCTCCGCCAAATCCAGACAA	GCTGCGTGCAATTCATACTTTTC
SETD4	GAAAGCTAGGAAGTTTCAAGATTCAAA	GACTCATCAGCCCTCTTCCTGTA
CBR1	CTGATCCCACACCCTTTCAT	TTAAGGGCTCTGACGCTCAT
CBR3	CCGTCGCCTTCAAGAGTGATG	CGGCAGTAACTCGTTGCACA
DOPEY2	TGAATGGGCGGATCTCATATCT	AGCCGTCTTGGCAACAAGG
MORC3	ATGCAGTATTGAGAGGGACCAGTAT	CACACTGTGAACGGATTTGTGA
CHAF1B	CCATCATATGGGATGTCAGCAA	CTTCATGCTGTCGTCGTGAAAC
HLCS	AGGACAAAGGGCCCAACAG	CGCTGCCCAGATGGACTT
DSCR6	GTCAAAGCGTCAAGAATACCTGC	AAGTCAATCGTGGCTTGCACT
PIGP	TGTTTGGGATTAACATGATGAGTACCT	TCTGCTGTTGATTTTTTGCATAGTT
TTC3	TCACTGTGGCGGATTATGCC	GTAAAGCTGAGTCACACGAACAT
DSCR3	CCTACACTGGAGACCACCAACTT	GATGAGGTGGTCAGGGTGAAG
DYRK1A	ACCACCTCCAGTACATCGTCAAG	CCGGGCTCTCCCACTGTT
ERG	GCTGCTCAACCATCTCCTTC	ACAGGAGCTCCAGGAGGAAC
ETS2	TGGAGACGGATGGGAGTTTA	CGACGTCTTGTGGATGATGT
WRB	CGTGCCGAGTAAATGGATAACC	GAAGCACAATAGCGACAACTTTG
MX1	GTTTCCGAAGTGGACATCGCA	CTGCACAGGTTGTTCTCAGC
NDUFV3	TCAGCCACAGAATTCCAAGAAG	CTGTAGTCATGATGCTGCAGGTT
CBS	GGCCAAGTGTGAGTTCTTCAA	GGCTCGATAATCGTGTCCCC
U2AF1	TGAACGTCTGTGACAACCTG	ATCACAGCCTTTTCCGCATC
CSTB	GCCACCGCCGAGACCCAGCA	TGGCTTTGTTGGTCTGGTAG
AGPAT3	GAGCCAACTGGTCATGCTG	ACCCACAGAGGAAGTCGATCT
PWP2	CCACTCGGTACAACGTCAAGT	TCAGGGGAGAAGGACACACTG
C21orf33	GATGCGGAGTCTACGATGGG	CCAGGTCTGTGATTTTGCCAC
PFKL	GGCATTTATGTGGGTGCCAAAGTC	CAGTTGGCCTGCTTGATGTTCTCA
SUMO3	GAATGACCACATCAACCTGAAGG	GCCCGTCGAACCTGAATCT
PTTG1IP	TGAAGAACGTCTCCTGTCTT	ACTACCGACATGGTGATGAT
FAM207A	GAGCGTCCCTTCCATCAGG	GCTCACGCCTCAGCTTCATT
COL6A	ACACCGACTGCGCTATCAAG	CGGTCACCACAATCAGGTACTT
C21orf56	TCCGGCCTAAGAAGGTGTGT	GGTGGAGGTCTGCTCGATCT
LSS	GCACTGGACGGGTGATTATGG	TCTCTTCTCTGTATCCGGCTG
YBEY	GCGCCACTTCGCAGTAAGAT	ACATCGGTTGGGACATTTCTATC
PCNT	AGGAGGAGAGTCCGGTAACC	TCAGGGGTGTCGTCACATGAT
PRMT2	GGATCATCAGTCTCTTCTGTGCA	GTGCTGTGCCATCTCACTGG
